# Health-related quality of life among women diagnosed with in situ or invasive breast cancer and age-matched controls: a population-based study

**DOI:** 10.1186/s41687-024-00781-1

**Published:** 2024-09-17

**Authors:** Synne K. H. Bøhn, Karianne Svendsen, A. Balto, Ylva Maria Gjelsvik, Tor Åge Myklebust, Elin Børøsund, Hege R. Eriksen, A. Meland, K. Østby, L. Solberg Nes, Cecilie E. Kiserud, Kristin V. Reinertsen, G. Ursin

**Affiliations:** 1 Unit for Late Effects after Cancer Treatment, Department of Oncology, Oslo University Hospital, Oslo, Norway; 2 Cancer Registry of Norway, Norwegian Institute of Public Health, Oslo, Norway; 3 The Lipid Clinic, Department of Endocrinology, Morbid Obesity and Preventive Medicine, Oslo University Hospital, Oslo, Norway; 4 Department of Nutrition, Institute of Basic Medical Sciences, Faculty of Medicine, University of Oslo, Oslo, Norway; 5 Department of Research and Innovation, Møre and Romsdal Hospital Trust, Ålesund, Norway; 6 Department of Digital Health Research, Division of Medicine, Oslo University Hospital, Oslo, Norway; 7 Department of Nursing and Health Sciences, Faculty of Health and Social Sciences, University of South-Eastern Norway, Drammen, Norway; 8 Department of Sport, Food and Natural Sciences, Western Norway University of Applied Sciences, Bergen, Norway; 9 Department of Social Sciences, Norwegian School of Sport Sciences, Oslo, Norway; 10 Department of Psychiatry and Psychology, Mayo Clinic College of Medicine and Science, Mayo Clinic, Rochester, MN USA; 11 Institute of Clinical Medicine, Faculty of Medicine, University of Oslo, Oslo, Norway

**Keywords:** Breast cancer, HRQoL, Fatigue, Late-effects, In-situ, Invasive, PROs, EORTC

## Abstract

**Purpose:**

A breast cancer (BC) diagnosis may negatively affect health-related quality of life (HRQoL). However, there are few comparisons of HRQoL at several time points for women with BC, and particular when subdivided into invasive and in situ tumors. The purpose of this study was to investigate various aspects of HRQoL in women recently diagnosed with invasive BC or ductal carcinoma in situ (in situ) compared to age-matched BC free controls in a population-wide sample recruited through the Cancer Registry of Norway.

**Methods:**

This cross-sectional study utilized HRQoL data collected in 2020–2022 from a digital survey including 4117 cases (3867 women with invasive BC and 430 with in situ) and 2911 controls. HRQoL was assessed ≥ 21 days after diagnosis, using EORTC QLQ-C30. This includes scores assessing global quality of life (gHRQoL) and HRQoL functions and symptoms. Multivariable regression analyses were used to compare HRQoL between cases and controls and to identify factors associated with gHRQoL and fatigue. Additionally, HRQoL 14 months after diagnosis was analyzed in 1989 of the included cases and in 1212 of the controls. Score differences of ≥ 10 points were considered clinically relevant and thus presented in the results.

**Results:**

Invasive BC cases had lower gHRQoL, role- and social functioning in addition to more fatigue than controls. In situ cases had lower role—and social functioning than controls. Invasive BC cases scored worse than in situ on all domains, but the differences were not considered clinically relevant. Physical activity was associated with better gHRQoL and less fatigue in invasive BC, in situ and controls. Both invasive BC and in situ cases improved their role- and social functioning scores from diagnosis to 14 months follow-up, however no improvement was seen for fatigue.

**Conclusion:**

Women with invasive BC and in situ reported lower role- and social functioning scores than controls right after diagnosis with improvements 14 months after diagnosis. Physical activity was associated with better gHRQoL and less fatigue and should, whenever possible, play a key role in the care for BC patients.

**Supplementary Information:**

The online version contains supplementary material available at 10.1186/s41687-024-00781-1.

## Background

Due to advances in diagnostics and treatments, the 5-year relative survival rate for women with breast cancer (BC) is steadily increasing and has exceeded 90% in several western countries [1, 2]. Effective BC treatment is often associated with adverse effects ranging from minor and transient, to serious and persistent conditions [3]. This again can cause reductions in functioning, social participation, and health-related quality of life (HRQoL) in the affected individuals [3–5]. HRQoL covers the subjective perceptions of physical, emotional, social, and cognitive functions as well as disease symptoms and side effects of treatment [6]. One of the most common and distressing symptoms during and after BC treatment is fatigue [7, 8]. Given the long life expectancy after BC, an important goal is to alleviate adverse effects during and after cancer treatment and to enhance patients’ HRQoL [3]. To optimize such care, early identification of those most at risk of poor HRQoL is important [9, 10]. Some studies have found differences across several HRQoL domains between individuals with invasive BC, ductal carcinoma in situ (in situ) and controls both shortly after diagnosis [11, 12], and several years after BC diagnosis [11, 13–15]. These differences seem to be more prominent for the physical health parameters, and less in the well-being or mental health parameters [12, 13].

A healthy lifestyle can have beneficial effects on several aspects of HRQoL in BC survivors [16, 17], however, exactly which factors play a role and how, is not clear. In the present study, we utilized national, patient reported outcomes (PROs) data on HRQoL collected between 2020 and 2022 in the Cancer Registry of Norway (CRN)’s HRQoL-Survey [18].

The aims were to compare HRQoL between women with invasive BC or in situ cancer in the breast shortly after diagnosis (baseline) and age-matched BC free controls, and to study changes in global HRQoL (gHRQoL), role- and social functioning and fatigue from baseline to 14-month follow-up. Further, we aimed to study the associations between lifestyle factors and HRQoL among women with invasive BC, in situ cases and controls.

## Material and methods

### The Cancer Registry of Norway and the Norwegian Breast Cancer Registry

The CRN receives comprehensive information on all cancer diagnoses from several sources including pathology reports, doctors’ registrations and hospitals [1]. Reporting of cancer cases and cases of certain precancerous conditions to the registry is mandatory by law, providing close to complete data [19]. The CRN administers the Norwegian Breast Cancer Registry, which contains detailed information on BC characteristics and treatment. Information on BC characteristics in the current study were obtained from these registries.

### The CRN HRQoL-Survey

The CRN has, since September 2020, administered the CRN HRQoL-Survey [18], an ongoing digital survey sent to individuals with different cancer types and age-matched controls. For BC, eligible cases are women aged ≥ 18 years when diagnosed with first occurrence of pathologically verified invasive BC (ICD10 = C50) or (ductal carcinoma) in situ (ICD10 = D05.9). According to the protocol, cases are invited from 21 days after date of the malignant biopsy to ensure that they are not invited to the survey before being informed about their BC diagnosis by their physician [18]. Potential controls are identified from the population registry and frequency matched to the distribution across age groups and region of residence of cases. Controls are randomly selected within these strata.

Individuals who use the official website for health communication in Norway (Helsenorge.no) or have an official digital mailbox (Digipost/e-box) are eligible and invited to the survey through this system. In 2020, the CRN were able to invite 79% of all individuals diagnosed with BC, whereas in 2022 the corresponding numbers were 86% [18]. The survey is available for 30 days, with a reminder sent to non-responders after 14 days.

At present, there are additionally two follow-up surveys sent to all women with BC. The first survey is sent about 14 months following diagnosis in BC cases (and on a similar date to controls), the second about 36 months following diagnosis.

In the present study, we included data on HRQoL measures from baseline (≥21 days following diagnosis), and also 14 months after diagnosis for a group of women diagnosed with BC and controls in a two-year period.

### Participants

**Fig. 1:**
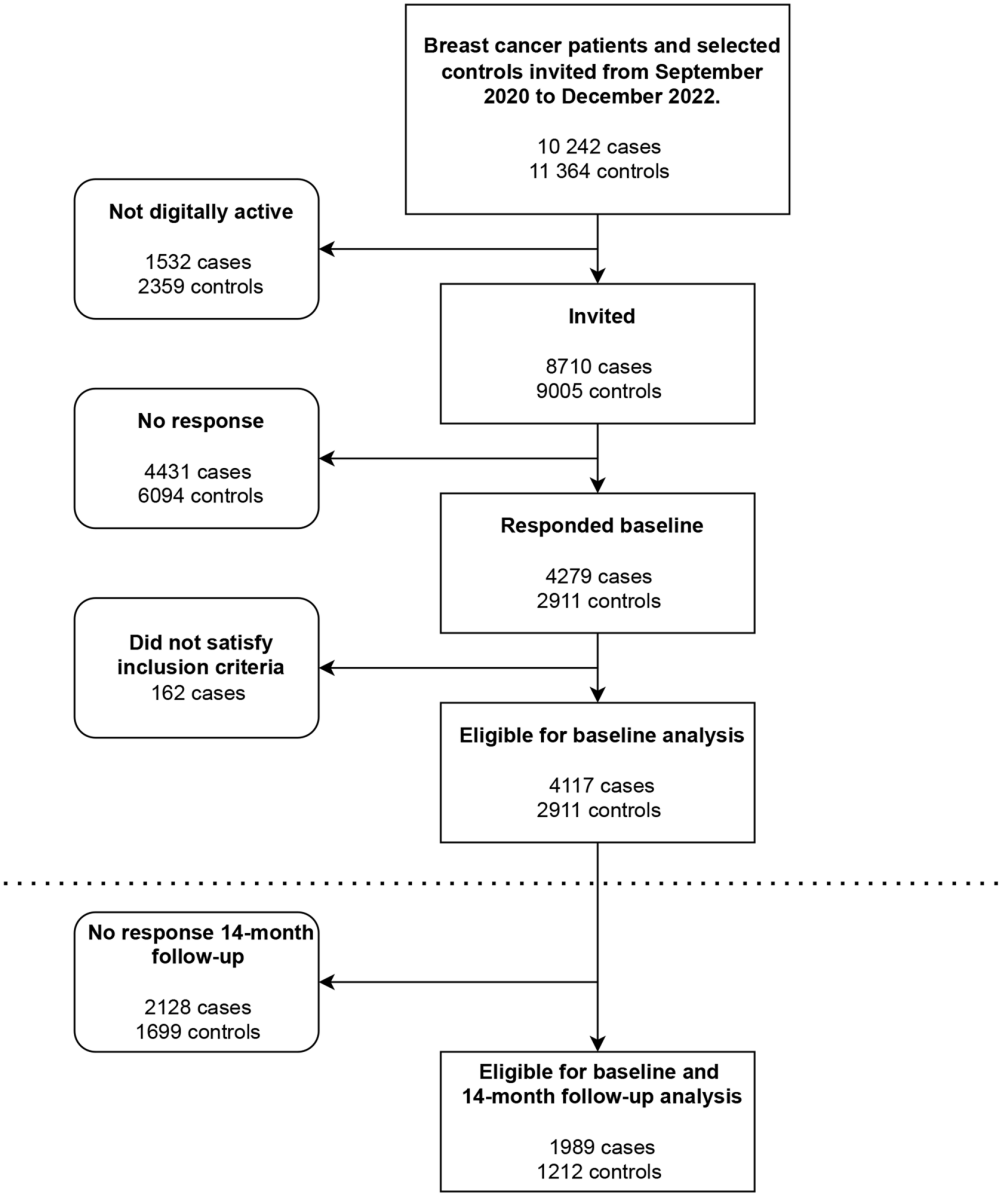
Flowchart of participant inclusion. Breast cancer patients and controls were invited from September 2020 to December 2022 (this includes breast cancer patients diagnosed from August 2020)

A total of 10,242 women diagnosed after August 1st, 2020, were invited between September 1st 2020 and December 31st, 2022. Among these cases, and the randomly sampled 11,364 age-matched controls, 8710 cases and 9005 controls could be invited digitally to the CRN HRQoL-Survey. A total of 4279 cases (49%) and 2911 controls (32%) responded. After more detailed review of the pathology records, we excluded 162 cases who did not have a malignant BC diagnosis during this period. Consequently, we included 4117 women with BC and 2911 controls in the analysis of baseline data. Of these, 1989 cases and 1212 controls also had data on the survey sent 14 months after diagnosis (Fig. 1).

### Variables

The CRN HRQoL-Survey largely adheres to the International Consortium for Health Outcomes Measurement (ICHOM) standards [10]. The survey includes (among others) the European Organization for Research and Treatment of Cancer Quality of Life 30-item Questionnaire (EORTC QLQ-C30) which has been widely used to assess HRQoL in oncological studies [20], and selected self-reported sociodemographic and lifestyle questions.

#### Health-related quality of life

EORTC QLQ-C30 includes a global health status and quality of life (gHRQoL) scale, functioning scales (physical, role, emotional, cognitive and social), symptoms scales (fatigue, nausea and vomiting, pain), other common cancer-related symptoms items (dyspnoea, insomnia, appetite loss, constipation, diarrhoea) and financial difficulties.

The EORTC QLQ-C30 scales ranging from 0 to 100 were calculated according to the EORTC QLQ-C30 scoring manual. At least 50% of the items within a scale must be answered to receive a score on that domain [20, 21]. High scores on gHRQoL and functional scales implies better gHRQoL and higher functioning [20]. High scores on symptom scales/items imply more, or worse, symptoms [20]. In the current study, a between group difference of ≥ 10 points or more in EORTC QLQ-C30 scale scores was considered clinically relevant [22].

#### Sociodemographic data

For this study, sociodemographic information included age at survey-response, relationship status (categorized as in a relationship versus not), level of education (primary school/secondary school/high school or university ≤ 4 years/high school or university > 4 years), and employment status assessed by the question “Are you employed?” and categorized as employed vs non-employed (including retired). Physical activity included information on both exercise (regular activities of higher intensity, such as running of at least 30 min duration) and light intensity activities such as walking, biking and gardening. These were combined as follows: “No exercise, but light activity ≤ 3 hours per week” (i.e physically inactive), “no exercise, but light activity > 3 hours per week”, “exercise 0–1 hour per week”, “exercise 2–3 hours per week” and “exercise ≥ 4 hours per week”. Smoking was assessed by the questions: “Do you smoke?” and “If no, have you smoked previously” (Yes/No), and based on this information, categorized into current, former or never smoker. Alcohol consumption was assessed by the question: “Do you drink alcohol?” (Yes/No). Body mass index (BMI, kg/m²) was calculated from the self-reported height (m) and weight (kg).

#### Medical data

BC characteristics (stage, tumour characteristics etc.), and information on surgery/radiotherapy in relation to survey response were obtained from the CRN. Information on systemic BC therapies were unavailable.

### Statistical analysis

Continuous variables were described by either means and standard deviations (SD) or median, minimum and maximum values (min-max), and categorical variables as numbers and percentages. Chi-square- and *t*-tests were used to compare differences between invasive BC, in situ and controls.

Multivariable linear regression models were used to compare mean differences in each of the HRQoL domains between groups at baseline. Univariable linear regression models were used to assess the change from baseline to 14 months after diagnosis for selected HRQoL domains. Multivariable linear regression models were also used to explore potential differences in associations between lifestyle, gHRQoL and fatigue separately in invasive BC, in situ cases and controls at baseline. All results are presented with 95% confidence intervals (95% CI). In all multivariable models, adjustments included the (a priori identified) potential confounding variables age (age groups), educational level, physical activity/exercise, smoking, alcohol use (yes/no) and BMI groups. To explore the strength of effects of each individual independent variable on gHRQoL and fatigue, we report the standardized β coefficients (std beta) from multivariable analysis. The std beta have standard deviations as their units, which allows for comparability across the set of independent variables [23].

All analyses were performed in Stata version 18.0 (StataCorp. 2023. Stata Statistical Software: Release 18. College Station, TX: StataCorp LLC.)

#### Sensitivity analyses

To address the influence of having surgery or radiation therapy, we conducted the analysis stratified by whether the cases had responded before or after surgery or radiation therapy. Women who did not have surgery at all were included in the “before surgery” category.

## Results

### Participant characteristics

Of the 4117 BC cases, 3867 (89.6%) were diagnosed with invasive BC, whereas 430 (10.4%) had in situ. Invasive and in situ BC cases were on average 58 years old at diagnosis and corresponding controls were 59 years. Most BC cases and controls had a partner, higher education and were employed. Lifestyle factors were similar among invasive BC, in situ and controls, with 13.5, 10.9 and 11.1% being inactive in the three groups. Mean BMI indicated slight overweight in all three groups (Table 1).

**Table 1:** Sociodemographic data on invasive breast cancer cases (*n* = 3867), ductal carcinoma in situ cases (in situ) (*n* = 430) and controls (*n* = 2911)

	Invasive BC (*n* = 3687)	In situ (*n* = 430)	Controls (*n* = 2911)	*P*-values invasive BC vs controls	*P*-values in situ vs controls
**Age at survey, mean (SD)**	58.2 (11.4)	58.2 (9.8)	59.1 (11.2)	<0.01	0.10
**In a relationship, *n*(%)**				0.88	0.67
No	726 (19.7)	90 (20.9)	575 (19.8)		
Yes	2845 (77.2)	331 (77.0)	2232 (76.7)		
Missing	116 (3.1)	9 (2.1)	104 (3.6)		
**Level of education, *n*(%)**				0.03	0.53
Primary school	272 (7.4)	27 (6.3)	238 (8.2)		
Secondary school	1301 (35.3)	150 (34.9)	1037 (35.6)		
High school/university ≤ 4 years	1067 (28.9)	138 (32.1)	890 (30.6)		
High school/university > 4 years	940 (25.5)	101 (23.5)	655 (22.5)		
Missing	107 (2.9)	14 (3.3)	91 (3.1)		
**Employed, *n*(%)**				0.61	0.47
No	1366 (37.0)	152 (35.3)	1058 (36.3)		
Yes	2150 (58.3)	266 (61.9)	1710 (58.7)		
Missing	171 (4.6)	12 (2.8)	143 (4.9)		
**Exercise, *n*(%)**				<0.01	0.06
No exercise, but light activity ≤ 3 h/w	496 (13.5)	47 (10.9)	323 (11.1)		
No exercise, but light activity > 3 h/w	1233 (33.4)	148 (34.4)	796 (27.3)		
Exercise 0–1 h per week	908 (24.6)	99 (23.0)	764 (26.2)		
Exercise 2–3 h per week	693 (18.8)	93 (21.6)	677 (23.3)		
Exercise ≥ 4 h per week	230 (6.2)	32 (7.4)	253 (8.7)		
Missing	127 (3.4)	11 (2.6)	98 (3.4)		
**Smoking, *n*(%)**				<0.01	0.51
Never smoker	1642 (44.5)	204 (47.4)	1366 (46.9)		
Former smoker	1589 (43.1)	173 (40.2)	1114 (38.3)		
Current smoker	351 (9.5)	42 (9.8)	334 (11.5)		
Missing	105 (2.8)	11 (2.6)	97 (3.3)		
**Drinks alcohol, *n*(%)**				<0.01	0.93
No	1026 (27.8)	101 (23.5)	683 (23.5)		
Yes	2553 (69.2)	319 (74.2)	2133 (73.3)		
Missing	108 (2.9)	10 (2.3)	95 (3.3)		
**Body mass index (kg/m**^**2**^**), mean (SD)**	26.1 (5.1)	25.5 (4.7)	25.8 (4.8)	<0.01	0.25
**EORTC QLQ-C30, mean (SD)**					
Functioning scales/quality of life*					
Global health status/quality of life	64.3 (21.2)	68.9 (21.2)	75.8 (20.3)	<0.01	<0.01
Physical functioning	83.2 (18.1)	86.3 (16.3)	88.4 (16.2)	<0.01	0.01
Role functioning	65.1 (30.7)	71.3 (32.0)	86.6 (23.1)	<0.01	<0.01
Emotional functioning	74.4 (20.9)	76.8 (21.4)	83.3 (17.9)	<0.01	<0.01
Cognitive functioning	80.9 (21.2)	84.1 (20.8)	86.3 (17.7)	<0.01	0.02
Social functioning	68.9 (26.1)	75.5 (26.6)	86.1 (22.7)	<0.01	<0.01
Symptom scales/single items**					
Fatigue	39.0 (25.2)	33.5 (25.0)	26.4 (21.7)	<0.01	<0.01
Nausea and vomiting	6.9 (12.8)	4.8 (11.9)	3.4 (9.1)	<0.01	0.01
Pain	27.4 (25.5)	24.5 (25.8)	22.7 (25.6)	<0.01	0.20
Dyspnoea	14.0 (22.0)	11.9 (20.5)	11.7 (20.5)	<0.01	0.89
Insomnia	33.8 (29.4)	30.0 (29.9)	27.7 (27.4)	<0.01	0.11
Appetite loss	15.0 (23.8)	10.8 (20.1)	5.9 (15.5)	<0.01	<0.01
Constipation	20.4 (27.8)	16.0 (24.4)	15.0 (23.6)	<0.01	0.40
Diarrhoea	13.8 (22.7)	10.6 (19.5)	13.9 (22.6)	0.93	<0.01
Financial difficulties	9.3 (21.1)	6.9 (19.0)	5.5 (17.0)	<0.01	0.11

*BC* breast cancer, *In situ* ductal carcinoma in situ, *SD* standard deviation, *h/w* hours per week. *EORTC QLQ-C30* European Organization for Research and Treatment of Cancer QLQ-C30

*Increasing score implies better functioning/quality of life

**Increasing score implies worse symptoms

In total 49% of invasive BC cases were diagnosed and included at stage 1, and the majority of invasive BC cases were ER+ or PR+ and HER2‒. The median number of days between date of diagnosis (from pathology reports) to survey response were 47 (18–483) for invasive BC and 48 (21–257) for in situ, of which 47 and 45% respectively responded ≤ 1 month after diagnosis. In total 28% of invasive BC cases and 14% of in situ cases responded prior to surgery (including those who did not have surgery at all). Those who had surgery at time of survey completion had most frequently breast conserving therapy alone or in combination with radiation therapy (Table 2).

**Table 2:** Medical data for invasive breast cancer cases (*n* = 3687) and ductal carcinoma in situ (in situ) cases (*n* = 430)

	Invasive BC (*n* = 3687)	In situ (*n* = 430)
**Stage, *n*(%)**		
I	1813 (49.2)	–
II	1067 (28.9)	–
III	312 (8.5)	–
IV	84 (2.3)	–
Unknown	411 (11.1)	430 (100.0)
**HER2-status, *n*(%)**		
Negative	3215 (87.2)	9 (2.1)
Positive	433 (11.7)	8 (1.9)
Unknown	39 (1.1)	413 (96.0)
**ER-status, *n*(%)**		
Negative	469 (12.7)	10 (2.3)
Positive	3184 (86.4)	56 (13.0)
Unknown	34 (0.9)	364 (84.7)
**PR-status, *n*(%)**		
Negative	1056 (28.6)	6 (1.4)
Positive	2596 (70.4)	8 (1.9)
Unknown	35 (0.9)	416 (96.7)
**Local treatment status at survey, *n*(%)**		
Responded prior to surgery*/radiation	1020 (27.7)	61 (14.2)
BCT	2101 (57.0)	293 (68.1)
BCT+radiation therapy	210 (5.7)	16 (3.7)
Mastectomy	340 (9.2)	59 (13.7)
Mastectomy+radiation therapy	7 (0.2)	0 (0.0)
Radiation therapy, surgery unknown	9 (0.2)	1 (0.2)
**Days from diagnosis to response, median (min-max)**	47.0 (18.0–483.0)	48.0 (21.0–257.0)
**Time from diagnosis to response, *n*(%)**		
≤ 1 month	1745 (47.3)	193 (44.9)
2–3 months	1886 (51.2)	220 (51.2)
4–6 months	49 (1.3)	16 (3.7)
> 6 months	7 (0.2)	1 (0.2)

*BC* breast cancer, *In situ* ductal carcinoma in situ, *HER2* Human Epidermal Growth Factor Receptor 2, *ER* estrogen receptor, *BCT* Breast conserving therapy

*or no surgery

### Differences in health-related quality of life in cases versus controls

Invasive BC cases had lower scores [adjusted mean difference (95% CI)] on gHRQoL: [−9.82 (−10.83, −8.82)], role functioning [−19.66 (−21.02, −18.30)] and social functioning [−15.26 (−16.46, −14.06)] compared to controls. Furthermore, they reported a higher fatigue score (indicating more fatigue) [10.80 (9.66, 11.94)] compared to controls.

In situ cases had lower scores on role functioning [−14.71 (−17.52, −11.91)] and social functioning [−10.22 (−12.70, −7.74)], and higher score on fatigue [7.03 (4.68, 9.39)] compared to controls.

Invasive BC cases had slightly worse HRQoL scores than in situ cases, with the largest difference seen for social functioning [−5.04 (−7.49, −2.59)] (Table 3).

**Table 3:** Difference in HRQoL measures among invasive BC and in situ cases and controls, adjusted for age, education level, smoking, physical activity, BMI and drinking alcohol

Domain	Invasive (*n*)	In situ (*n*)	Control (*n*)	Invasive BC vs control Adjusted mean difference (95% CI)	In situ vs control Adjusted mean difference (95% CI)	Invasive BC vs in situ Adjusted mean difference (95% CI)
Global health status/quality of life*	3473	403	2719	−9.82 (−10.83, −8.82)	−6.80 (−8.87, −4.73)	−3.02 (−5.07, −0.98)
Functioning scales*						
Physical functioning	3470	404	2719	−4.05 (−4.85, −3.24)	−2.65 (−4.31, −0.99)	−1.40 (−3.03, 0.24)
Role functioning	3468	404	2719	−19.66 (−21.02, −18.30)	−14.71 (−17.52, −11.91)	−4.95 (−7.71, −2.18)
Emotional functioning	3468	404	2716	−7.91 (−8.88, −6.93)	−6.67 (−8.69, −4.65)	−1.24 (−3.22, 0.75)
Cognitive functioning	3468	404	2717	−4.27 (−5.24, −3.29)	−2.27 (−4.28, −0.27)	−1.99 (−3.97, −0.01)
Social functioning	3469	404	2715	−15.26 (−16.46, −14.06)	−10.22 (−12.70, −7.74)	−5.04 (−7.49, −2.59)
Symptom scales**						
Fatigue	3470	403	2716	10.80 (9.66, 11.94)	7.03 (4.68, 9.39)	3.76 (1.44, 6.09)
Nausea and vomiting	3469	404	2716	2.99 (2.43, 3.55)	1.26 (0.11, 2.41)	1.73 (0.60, 2.87)
Pain	3469	404	2719	3.14 (1.87, 4.41)	1.76 (−0.86, 4.37)	1.38 (−1.20, 3.96)
Dyspnoea	3463	403	2715	1.39 (0.34, 2.44)	0.93 (−1.23, 3.10)	0.46 (−1.6, 2.60)
Insomnia	3467	404	2712	5.15 (3.72, 6.59)	1.81 (−1.16, 4.77)	3.35 (0.42, 6.27)
Appetite loss	3462	403	2711	8.21 (7.20, 9.22)	5.05 (2.96, 7.14)	3.16 (1.10, 5.22)
Constipation	3459	403	2708	4.45 (3.14, 5.76)	1.26 (−1.44, 3.95)	3.19 (0.53, 5.85)
Diarrhea	3455	402	2714	−0.70 (−1.82, 0.43)	−2.95 (−5.28, −0.62)	2.25 (−0.05, 4.55)
Financial difficulties	3462	403	2706	2.86 (1.92, 3.81)	1.47 (−0.49, 3.43)	1.39 (−0.54, 3.32)

*In situ* ductal carcinoma in situ, *95% CI* 95% confidence interval, *BC* breast cancer

*negative values imply worse functioning among cases compared to controls or invasive compared to DCIS

**positive values imply worse symptoms among cases (invasive and in situ) compared to controls

All adjustment variables are categorical with categories as seen in Table 1

### Difference from baseline to 14 months after diagnosis

From baseline to 14 months after diagnosis, both invasive BC and in situ cases experienced improvements in gHRQoL, role- and social functioning. The HRQoL scores for controls were largely unchanged from baseline to 14 months after diagnosis. The largest improvements were observed for in situ cases, particular for role-functioning [mean difference 13.9 (9.5, 18.3)]. There was little difference in the fatigue scores for all three groups (Table 4).

**Table 4:** Unadjusted mean and change in HRQoL measures from baseline to 14-months follow-up among invasive breast cancer cases, ductal carcinoma in situ (in situ) cases and controls

	Invasive BC (*n* = 1820)	In situ (*n* = 169)	Control group (*n* = 1212)
	Mean (95% CI)	Mean (95% CI)	Mean (95% CI)
**Global health status/quality of life**			
Baseline	65.11 (64.15, 66.06)	67.74 (64.63, 70.85)	77.29 (76.12, 78.46)
14 months follow-up	66.30 (65.33, 67.27)	72.75 (69.61, 75.89)	75.96 (74.78, 77.14)
Change*	1.20 (0.29, 2.10)	5.01 (2.06, 7.96)	−1.33 (−2.44, −0.22)
**Role functioning**			
Baseline	65.84 (64.53, 67.15)	68.40 (64.17, 72.64)	87.79 (86.19, 89.39)
14 months follow-up	73.87 (72.65, 75.09)	82.31 (78.36, 86.26)	86.52 (85.03, 88.01)
Change*	8.03 (6.68, 9.38)	13.91 (9.54, 18.27)	−1.27 (−2.91, 0.38)
**Social functioning**			
Baseline	69.53 (68.38, 70.67)	75.56 (71.85, 79.27)	87.58 (86.18, 88.98)
14 months follow-up	71.87 (70.67, 73.07)	79.04 (75.15, 82.93)	86.18 (84.71, 87.64)
Change*	2.34 (1.22, 3.47)	3.48 (−0.16, 7.12)	−1.40 (−2.77, −0.02)
**Fatigue**			
Baseline	37.76 (36.65, 38.87)	34.74 (31.13, 38.35)	24.49 (23.14, 25.85)
14 months follow-up	39.85 (38.71, 40.99)	33.40 (29.69, 37.11)	27.01 (25.61, 28.41)
Change**	2.10 (1.08, 3.11)	−1.34 (−4.65, 1.97)	2.52 (1.27, 3.76)

*BC* breast cancer, *CI* confidence interval

*Positive values indicate better quality of life/functioning, while negative values indicate worse quality of life/functioning

**Negative values indicate less fatigue, while positive values indicate more fatigue

Baseline = At least 21 days following breast cancer diagnosis

### Lifestyle factors associated with gHRQoL

In invasive BC cases, weekly physical activity was associated with clinically relevant higher scores on gHRQoL, with the highest std beta (0.26) observed for exercise 2–3 times a week. A positive association between gHRQoL and some (versus totally avoiding) alcohol consumption with std beta 0.08 was also observed. Additionally, age groups ≥ 50 years were associated with higher scores on gHRQoL with std beta ≥ 0.15, while former smoking was associated with lower gHRQoL (-std beta −0.04).

The same positive associations for physical activity and drinking alcohol were seen for in situ cases with highest std beta 0.32 for exercise ≥ 4 times a week, and 0.14 for drinking alcohol. Contrary, former smoking (std beta-0.16) and higher body mass index (std beta −0.11 and −0.10 for overweight and obesity respectively) were negatively associated with gHRQoL. Compared to the lowest education level, higher educational level was associated with lower gHRQoL in in situ cases, but with wide confidence intervals.

The results for controls followed the same pattern as for cases for physical activity with highest std beta (0.23) for exercise ≥ 4 times. Age ≥ 50 years was positively associated with gHRQoL (std beta 0.14), whereas overweight/obesity and former/current smoking was negatively associated with gHRQoL (std beta −0.06/−0.14 and −0.06/−0.10) (Table 5).

**Table 5:** Multivariable linear regression analyses with global quality of life (global health status/quality of life) as dependent variable within invasive breast cancer (BC) cases, ductal carcinoma in situ (in situ) cases and controls

	Invasive BC cases (*n* = 3473)	In situ (*n* = 403)	Controls (*n* = 2719)	Invasive BC cases	In situ	Controls
Predictors	Regression coefficient (95% CI)			Standardized beta coefficients		
Age						
18–49 years	Ref.	Ref.	Ref.	Ref.	Ref.	Ref.
50–69 years	6.68 (4.99, 8.37)	4.23 (−1.56, 10.02)	5.87 (3.97, 7.77)	0.15	0.08	0.14
≥ 70 years	10.89 (8.64, 13.14)	1.05 (−7.23, 9.34)	4.43 (1.92, 6.94)	0.19	0.01	0.08
Education						
Primary school	Ref.	Ref.	Ref.	Ref.	Ref.	Ref.
Secondary school	0.99 (−1.72, 3.70)	−8.42 (−16.88, 0.04)	0.71 (−2.11, 3.54)	0.02	−0.19	0.02
High school/university ≤ 4 years	−1.04 (−3.83, 1.75)	−10.34 (−19.01, −1.67)	0.57 (−2.35, 3.49)	−0.02	−0.23	0.01
High school/university > 4 years	−0.70 (−3.59, 2.20)	−15.18 (−24.21, −6.15)	1.22 (−1.84, 4.27)	−0.01	−0.31	0.03
Exercise						
No exercise, but light activity ≤ 3 h/w	Ref.	Ref.	Ref.	Ref.	Ref.	Ref.
No exercise, but light activity > 3 h/w	8.86 (6.69, 11.02)	9.62 (2.59, 16.66)	5.96 (3.38, 8.53)	0.20	0.22	0.13
Exercise 0–1 h per week	10.43 (8.14, 12.72)	14.53 (7.03, 22.02)	6.95 (4.34, 9.56)	0.22	0.29	0.15
Exercise 2–3 h per week	14.00 (11.55, 16.45)	14.43 (6.73, 22.13)	9.23 (6.54, 11.91)	0.26	0.28	0.19
Exercise ≥ 4 h per week	16.84 (13.54, 20.14)	25.21 (15.72, 34.69)	16.45 (13.12, 19.79)	0.20	0.32	0.23
Smoking						
Never smoker	Ref.	Ref.	Ref.	Ref.	Ref.	Ref.
Former smoker	−1.80 (−3.28, −0.33)	−6.89 (−11.25, −2.52)	−2.52 (−4.12, −0.91)	−0.04	−0.16	−0.06
Current smoker	−0.61 (−3.05, 1.83)	−2.75 (−10.27, 4.77)	−6.08 (−8.51, −3.64)	−0.01	−0.04	−0.10
Drinks alcohol						
No	Ref.	Ref.	Ref.	Ref.	Ref.	Ref.
Yes	3.70 (2.15, 5.25)	7.20 (2.37, 12.04)	4.78 (3.04, 6.52)	0.08	0.14	0.10
Body mass index						
< 25 kg/m^2^	Ref.	Ref.	Ref.	Ref.	Ref.	Ref.
25–29 kg/m^2^	−0.59 (−2.13, 0.96)	−5.04 (−9.61, −0.46)	−2.68 (−4.33, −1.02)	−0.01	−0.11	−0.06
≥ 30 kg/m^2^	−2.71 (−4.55, −0.87)	−5.18 (−10.64, 0.28)	−7.18 (−9.22, −5.15)	−0.05	−0.10	−0.14

*In situ* intraductal carcinoma in situ, *95% CI* 95% confidence interval, *Ref* reference (0.00). Regression coefficients adjusted for age, educational level, physical activity/exercise, smoking, alcohol use (yes/no) and BMI groups

### Lifestyle factors associated with fatigue

Among invasive BC, higher levels of weekly physical activity and higher age (≥50 years) were inversely associated with fatigue, with the highest std beta (−0.25) for exercise 2–3 h per week. Drinking alcohol (compared to totally avoiding) was also associated with less fatigue with std beta −0.09. Former smoking was associated with more fatigue.

Among women with in situ, associations were similar. The highest std beta (−0.28) was observed for exercise > 4 h a week. Drinking alcohol was associated with less fatigue (std beta −0.13), whereas former smoking and overweight (compared to normal weight) were associated with more fatigue (std beta 0.17 and 0.15, respectively).

For controls, increasing physical activity levels were associated with less fatigue, with highest std beta for exercise > 4 h a week (−0.21). Age 50–69 and alcohol consumption were also associated with less fatigue among controls, while overweight/obesity and former/current smoking were associated with more fatigue (Table 6).

**Table 6:** Multivariable linear regression analysis with fatigue score as dependent variable among invasive breast cancer (BC) cases, ductal carcinoma in situ cases (in situ) and controls

	Invasive BC cases (*N* = 3470)	In situ (*N* = 403)	Controls (*N* = 2716)	Invasive BC cases	In situ cases	Controls
Predictors	Regression coefficient (95% CI)			Standardized beta coefficients		
Age						
18–49 years	Ref.	Ref.	Ref.	Ref.	Ref.	Ref.
50–69 years	−11.36 (−13.36, −9.35)	−5.80 (−12.64, 1.04)	−7.70 (−9.74, −5.65)	−0.22	−0.10	−0.17
≥ 70 years	−16.68 (−19.34, −14.02)	−7.98 (−17.78, 1.82)	−6.61 (−9.31, −3.91)	−0.24	−0.09	−0.11
Education						
Primary school	Ref.	Ref.	Ref.	Ref.	Ref.	Ref.
Secondary school	0.45 (−2.76, 3.66)	1.32 (−8.69, 11.33)	−1.12 (−4.17, 1.92)	0.01	0.03	−0.02
High school/university ≤ 4 years	0.73 (−2.58, 4.03)	4.76 (−5.49, 15.01)	0.27 (−2.88, 3.41)	0.01	0.09	0.01
High school/university > 4 years	1.96 (−1.46, 5.38)	10.72 (0.07, 21.37)	−0.36 (−3.65, 2.93)	0.03	0.18	−0.01
Exercise						
No exercise, but light activity ≤ 3 h/w	Ref.	Ref.	Ref.	Ref.	Ref.	Ref.
No exercise, but light activity > 3 h/w	−10.17 (−12.73, −7.61)	−8.50 (−16.81, −0.18)	−5.05 (−7.81, −2.28)	−0.19	−0.16	−0.10
Exercise 0–1 h per week	−12.55 (−15.26, −9.84)	−16.20 (−25.07, −7.34)	−6.80 (−9.60, −3.99)	−0.22	−0.27	−0.14
Exercise 2–3 h per week	−16.13 (−19.03, −13.23)	−9.58 (−18.69, −0.48)	−9.19 (−12.07, −6.30)	−0.25	−0.16	−0.18
Exercise ≥ 4 h per week	−19.32 (−23.23, −15.42)	−25.81 (−37.03, 14.58)	−15.74 (−19.32, −12.16)	−0.19	−0.28	−0.21
Smoking						
Never smoker	Ref.	Ref.	Ref.	Ref.	Ref.	Ref.
Former smoker	2.25 (0.50, 4.00)	8.56 (3.40, 13.72)	2.51 (0.78, 4.24)	0.04	0.17	0.06
Current smoker	0.39 (−2.50, 3.28)	8.50 (−0.34, 17.34)	4.80 (2.17, 7.42)	0.00	0.10	0.07
Drinks alcohol						
No	Ref.	Ref.	Ref.	Ref.	Ref.	Ref.
Yes	−5.27 (−7.10, −3.44)	−7.63 (−13.32, −1.95)	−6.53 (−8.40, −4.65)	−0.09	−0.13	−0.13
Body mass index						
< 25 kg/m^2^	Ref.	Ref.	Ref.	Ref.	Ref.	Ref.
25–29 kg/m^2^	0.15 (−1.68, 1.98)	8.11 (2.71, 13.52)	2.14 (0.35, 3.92)	0.00	0.15	0.05
≥ 30 kg/m^2^	0.97 (−1.21, 3.14)	5.88 (−0.58, 12.34)	5.54 (3.35, 7.74)	0.02	0.09	0.10

*In situ* ductal carcinoma in situ, *95% CI* 95% confidence interval, *Ref* reference (0.00). Regression coefficients adjusted for age, educational level, physical activity/exercise, smoking, alcohol use (yes/no) and BMI groups

#### Sensitivity analyses

We compared HRQoL differences between those who responded to the survey prior to surgery and/or radiation therapy or did not have surgery/radiation therapy (*n* = 1020 invasive and *n* = 61 in situ), to those who had received surgery or radiation therapy at time of survey response (*n* = 2667 invasive, *n* = 369 in situ). Except for role functioning, the case-control differences were somewhat larger *prior to* surgery than after surgery. However, the differences were generally small, except for the case-control difference for social functioning which was larger for those who responded prior to surgery −19.4 (−21.1, −17.6) compared to after surgery −13.4 (−14.6, −12.2) (Supplementary File 1).

## Discussion

### Main findings

We found that invasive BC cases as well as those with in situ tumours had clinically relevant lower scores on role- and social functioning right after diagnosis compared with controls. Invasive BC cases also had lower gHRQoL and more fatigue. The role- and social functioning scores of invasive BC and in situ cases were higher at 14 months after diagnosis, but there was no change in fatigue scores. Physical activity was the strongest predictor for high HRQoL and most strongly inverse predictor of fatigue among both cases and controls.

#### HRQoL in invasive BC and in situ cases and controls

The clinically relevant lower scores of role- and social functioning for both invasive and in situ cases are in line other studies on BC patients demonstrating differences across several HRQoL domains about 6 weeks after diagnosis [11]. Invasive BC cases had worse HRQoL than in situ cases, in line with other studies [12, 13, 24], however no between-group differences were considered clinically relevant. Most invasive BC cases in our study were diagnosed at stage I and II, thus lower HRQoL scores could have been excepted if we ought to include more advanced stage BC patients [25].

We suspect that the disruptions found in several domains such as role- and social functioning in both invasive BC and in situ cases compared to controls partly reflect the negative effects of BC therapies on these women’s ability to work (58% of invasive BC cases and 62% of in situ cases were employed) and participate in everyday activities [26]. Results emphasizing lower social functioning in women with BC compared to controls have been demonstrated in other studies [27, 28] and is associated with pessimism [27, 28]. One might suspect that in general, individuals with more pessimistic personality traits might seek and have less social support available, leaving them at greater risk of experiencing low overall HRQoL. Reasuringly, both invasive BC and in situ cases had significant improvements in role- and social functioning from baseline to 14 months after diagnosis, in line with other studies in BC survivors [15, 29]. The improvements were largest for in situ cases, indicating they to a larger extent than invasive BC cases are “back to normal”, in terms of leisure activities etc., a little more than 1 year after diagnosis. In the Pink SWAN study, BC survivors had worse HRQoL at diagnosis and 1-year follow up compared to the control group, however after 2 years there was no significant difference between the groups [14]. It remains to be elucidated if a similar pattern is observed at the 36-month follow-up of the CRN HRQoL-Survey.

About 20–30% of BC survivors experience persistent fatigue up to 10 years after diagnosis [7, 8]. We observed a slightly higher fatigue score in invasive BC than in situ cases shortly after diagnosis, with somewhat larger case-control difference in those who responded prior to surgery or radiation therapy (adjusted mean difference 12.9 (11.3, 14.6) compared to post surgery (9.6 (8.5, 10.8). This may suggest that the fear of BC during the diagnostic work-up may already have induced fatigue and negatively impacted their HRQoL at the time of diagnosis. Moreover, there was minimal change in the fatigue scores from baseline to 14 months after diagnosis for both invasive BC and in situ cases, supporting that fatigue may persist long beyond the main treatment period [15, 27, 30–32]. Consequently, assessing fatigue symptoms and early identification of those most at risk of low HRQoL is paramount to offer early effective intervention and optimized follow-up aiming to hinder persistent low HRQoL in women diagnosed with BC. Such interventions, including psychosocial stress management interventions, psychoeducational support and physical rehabilitation [33, 34], are effective to mitigate the negative consequences of diagnosis and treatment [14, 27, 32–35]. Our study points to close follow-up of the HRQoL among women with invasive BC shortly after diagnosis for several HRQoL measures with additional long-term follow-up of both invasive BC and in situ cases when it comes to fatigue. In Norway, implementation of “standardized pathway HOME” is ongoing, exactly aiming to improve this part of cancer care [36].

#### Lifestyle factors affecting gHRQoL and fatigue

Being physically active was associated with better gHRQoL and less fatigue in invasive BC and in situ cases, in line with other studies in this patient group [16, 17, 37]. This study thus emphasizes physical activity as an important, low threshold self-management strategy for BC patients [3, 38]. The evidence regarding specific types or doses of physical activity unique for BC survivors is however limited [37]. We found stronger associations with HRQoL for exercising 2–4 h week (compared to less) in both BC cases and controls, and for invasive BC cases and controls the results indicated that “the more the exercise the better”. This observation is supported by a study showing that low physical activity is associated with HRQoL deterioration [39].

The association between lifestyle factors and HRQoL seemed to be stronger for in situ cases than invasive BC cases, indicating that the severity of the disease may impact lifestyle factors which again impacts HRQoL [25]. Nevertheless, we found that drinking alcohol was associated with better gHRQoL and less fatigue among both cases and controls. These findings are in agreement with a Chinese study, which reported that drinking alcohol was associated with better HRQoL among more than 1000 women diagnosed with BC within the last two weeks [40]. The apparently positive effects of alcohol could be due to moderate alcohol intake in social settings [41, 42]. Besides lifestyle factors, the negative association between higher education and gHRQoL was surprising, however there were only 27 women in the lowest education group so these results may be a result of random variation.

The survey did not assess dietary factors, but previous studies have shown that a healthy dietary pattern also has positive effects on HRQoL [43], and should therefore be part of the recommended lifestyle in this patient group. Emphasize on continued healthy lifestyle beyond diagnosis and treatment is important, as recent evidence indicates that insufficient physical activity, excess body weight and smoking predict worsening of HRQoL the next years [39, 44].

### Strengths and limitations

The main strength of this study is the nationwide sample of women with BC recruited through CRN, yielding a large sample size, and avoiding the potential biases arising from recruiting through cancer centers or restricted geographical areas. This solely digital CRN HRQoL-survey reached ≥ 79% of all women diagnosed with BC diagnosis in Norway after 2020 [45]. The survey largely consists of well-established and validated PROs that allow us to assess the effects of many factors simultaneously. The CRN started sending surveys to BC patients in late September 2020, therefore all surveys were completed *after* the start of the pandemic, and we have previously demonstrated, in the same sample of BC survivors and controls, that the case-control differences were similar across different COVID-19 phases [46].

The current study also has limitations. We do not have information on systemic BC treatment, but there are national guidelines and adherence to these are monitored through the Norwegian Breast Cancer Registry [45]. HRQoL and lifestyle factors were self-reported, which are subject to errors. Our response rate was moderate, 49% among cases and 32% among controls. Although the response rate in BC cases was similar or better than two postal PROs-surveys in Ireland and Australia using cancer registries as sampling frames [47, 48], we cannot exclude the possibility of response bias affecting our results. Furthermore, both cases and controls have on average higher education than the general population [49]. Finally, even if no response bias, generalization of the results may be limited to Norwegian women who are digitally active (80–90%).

## Conclusion

Invasive BC cases had worse HRQoL measures than controls and in situ BC cases shortly after diagnosis with improvements observed 14 months later. The fatigue scores were however not improved 14 months after diagnosis, and thus early identification and consequently interventions to improve fatigue in BC survivors is warranted. Early interventions should include physical activity as this was the strongest inverse predictor of fatigue, and the largest contributor to better gHRQoL among invasive BC and in situ cases and controls.

## Electronic supplementary material

Below is the link to the electronic supplementary material.

Supplementary Material 1

## Data Availability

Raw data were generated at CRN. Derived data supporting the findings of this study are available from the corresponding author [KS] on request.
