# Supplementary material for: Health-related quality of life among women diagnosed with in situ or invasive breast cancer and age-matched controls: a population-based study
Source: J Patient Rep Outcomes. 2024 Sep 17;8:105. doi: 10.1186/s41687-024-00781-1 (PMC11413291; doi:10.1186/s41687-024-00781-1)
Supplement: Supplementary file 1 — Supplementary Material 1 [file 41687_2024_781_MOESM1_ESM.docx]

**Supplementary file 1:** Differences HRQoL measures compared to controls (n=2719) in cases who responded prior to treatment (n=3739) or after surgery/radiation therapy (n=5575).

|  | **Responded prior to surgery (N=3739)** | **Responded after surgery (N=5575)** |
| --- | --- | --- |
| **Domain** | Adjusted mean difference (95%CI) | Adjusted mean difference (95%CI) |
| Global health status/quality of life* | -10.10 (-11.58 , -8.62) | -9.45 (-10.49 , -8.42) |
| *Functioning scales** |  |  |
| Physical functioning | -4.94 (-6.11 , -3.77) | -3.74 (-4.56 , -2.92) |
| Role functioning | -18.02 (-19.90 , -16.14) | -19.86 (-21.25 , -18.48) |
| Emotional functioning | -9.00 (-10.40 , -7.60) | -7.48 (-8.48 , -6.48) |
| Cognitive functioning | -19.38 (-21.14 , -17.62) | -13.38 (-14.61 , -12.15) |
| Social functioning | -6.60 (-7.99 , -5.21) | -3.33 (-4.32 , -2.33) |
| *Symptom scales/single items*** |  |  |
| Fatigue | 12.95 (11.30 , 14.59) | 9.62 (8.46 , 10.78) |
| Nausea and vomiting | 6.49 (5.68 , 7.31) | 1.64 (1.11 , 2.17) |
| Pain | -1.22 (-3.11 , 0.67) | 4.56 (3.25 , 5.87) |
| Dyspnoea | 5.29 (3.73 , 6.86) | 0.01 (-1.05 , 1.06) |
| Insomnia | 6.39 (4.30 , 8.47) | 4.48 (2.99 , 5.97) |
| Appetite loss | 12.37 (10.96 , 13.78) | 6.53 (5.55 , 7.50) |
| Constipation | 11.35 (9.39 , 13.31) | 1.71 (0.43 , 2.99) |
| Diarrhea | 3.83 (2.08 , 5.58) | -2.48 (-3.62 , -1.34) |
| Financial difficulties | 4.87 (3.46 , 6.28) | 2.03 (1.10 , 2.97) |

CI: confidence interval. *negative values imply worse functioning among cases compared to controls. **positive values imply worse symptoms among cases compared to controls. The prior to surgery group also includes those who did not have surgery at all. Adjustments included age (age groups), educational level, physical activity/exercise, smoking, alcohol use (yes/no) and BMI groups
